# Trends of Adverse Events Following Immunization (AEFI) Reports of Human Papillomavirus Vaccine in the Valencian Community—Spain (2008–2018)

**DOI:** 10.3390/vaccines8010117

**Published:** 2020-03-02

**Authors:** Cecilia M. Egoavil, José Tuells, Juan José Carreras, Emilia Montagud, Eliseo Pastor-Villalba, Pablo Caballero, Andreu Nolasco

**Affiliations:** 1 Hospital General Universitario de Alicante, Unit of Clinical Pharmacology, 03010 Alicante, Spain; mayaego1@gmail.com; 2 Department of Community Nursing, Preventive Medicine and Public Health and History of Science, University of Alicante, San Vicente del Raspeig, 03690 Alicante, Spain; pablo.caballero@ua.es (P.C.); nolasco@ua.es (A.N.); 3 Centro de Farmacovigilancia de la Comunidad Valenciana, Dirección General de Farmacia y Productos Sanitarios, Conselleria de Sanitat Universal i Salut Pública, 46010 Valencia, Spain; carreras_jua@gva.es; 4 Hospital Universitario del Vinalopó, Elche, 03293 Alicante, Spain; emontagud@vinaloposalud.com; 5 Dirección General de Salud Pública y Adicciones. Conselleria de Sanitat Universal i Salut Pública, 46021 Valencia, Spain; pastor_eli@gva.es

**Keywords:** adverse events following immunization (AEFIs), Human Papillomavirus vaccines, Spanish Pharmacovigilance System, vaccine safety, postlicensure surveillance

## Abstract

Vaccine safety surveillance is essential in vaccination programs. We accomplished a descriptive study of surveillance AEFI-reporting rate in human papillomavirus (HPV) vaccine administered in the Valencian Community, Spain. Data were obtained from Spanish Pharmacovigilance Adverse Reactions Data (FEDRA). Reporting rates were calculated using local net doses distributed as the denominator. Trends were assessed using joinpoint regression with annual percent change (APC) reported. The AEFI-reports decreased between 2008 and 2018 in two periods, a fast decreasing rate from 2009 to 2011 (from 192.2 to 24.93 per 100000 doses; APC, −54.9%; 95%CI [−75.2; −17.7]), followed by a stable trend (−13% APC, 95%CI [−26.1; 2.4]). For the age group analysis, only the group aged 14–15 years old followed the same trend with -58.4% (95%CI [−73.9; −33.8]) APC during 2008–2011, and −8.8% (95%CI [−27.7; 15]) APC during 2011-2018. The majority of the reports (73.82%) were nonserious, involving reactions at or near the vaccination site, headache, and dizziness events. No death was reported. AEFI-reporting rates for HPV immunization in the Valencian Community have decreased considerably with two trend periods observed for girls aged 14–15 years old. Currently, the AEFI reporting rate shows a decreasing trend, perhaps following the Weber effect, and it could also be affected by media attention and coverage.

## 1. Introduction

The World Health Organization recommends the human papillomavirus (HPV) vaccine for the prevention of cervical cancer and other diseases preventable by HPV vaccination [1]. 

The introduction of HPV vaccines into childhood vaccination programs shows compelling evidence of their substantial impact on HPV infection rates and the resulting fewer cases of genital warts, also providing excellent protection against cervical intraepithelial neoplasia among the population [2,3], and in situ adenocarcinoma associated with HPV16/18 infection in young women who were not initially infected [4]. Further, programs with multicohort vaccination and high vaccination coverage have had a greater and direct impact and positive herd effects [3]. 

The safety and tolerability of each HPV vaccine have been studied extensively in prelicensure clinical trials with similar profiles in the vaccinated and control groups, irrespective of age or ethnicity [5,6,7,8,9,10]. However, as the risk perception of diseases preventable by HPV decreases because of successful immunization programs, the risk perceived of the adverse events following immunization (AEFI) may increase [11], and for these reasons, postlicensure vaccine safety monitoring and evaluation programs play an important role in maintaining the public trust and the support of vaccination programs [12]. 

Adverse event reports have been collected and pooled into large databases in different countries in order to identify rare safety concerns in a timely fashion [13]. Spain has a national health system with universal access funded from general taxation; it is decentralized, and the country’s 19 regions (17 Autonomous Communities and two Autonomous Cities) are responsible for the management and delivery of vaccination programs [14]. Currently in Spain, three HPV vaccines are available: a bivalent (bHPV), a quadrivalent (qHPV), and a nonavalent vaccine (nHPV). 

The Ministry of Health leads and coordinates the Interterritorial Council and the Commission on Public Health. The Interterritorial Council is Spain’s main decision-making institution for the coordination of all vaccine programs and the Commission on Public Health and makes recommendations or proposes public health programs [14]. In September 2007, the HPV vaccination program was approved by the Commission on Public Health and by the Interterritorial Council in October that same year [15]. The Autonomous Communities implemented the HPV vaccine during 2007–2008, with some choosing the qHPV vaccine (Gardasil) and others the bHPV vaccine (Cervarix) [14].

In October 2008, the HPV vaccine was implemented in the Valencian Community immunization programs for females aged 14 years old with three doses of the qHPV vaccine (Gardasil) [16] and was administered in healthcare centers and schools [14]. 

In 2011, this authorization was modified and the Valencian Community immunization programs changed the qHPV vaccine (Gardasil) for the bHPV vaccine (Cervarix) [17]. In 2015, a new modification, the reduction of doses, was introduced, and the vaccination was extended to girls aged 12 years old [16,18]. 

The Valencian Community has about five million inhabitants [19] and a successful vaccination program with high vaccination coverage, and in 2019, this was estimated to be 87.7% for girls born in 2006 for the first dose, and 73.4% for the second dose, showing a slight decrease compared to previous years [20]. 

In February 2009, there were two reported cases of status epilepticus with myoclonus in two girls after the administration of the second dose of the qHPV vaccine (Gardasil) at schools in the same city of the Valencian Community [14,21]. Those cases were extensively covered by all media at the regional and national levels [22]. Subsequently, in 2013, a study described the suspected adverse reactions following the HPV vaccine during the first four years of HPV vaccination [23]. 

The objective of this study is to summarize the trends in rates of adverse events attributable to HPV vaccine use in the Valencian Community from January 2008 to December 2018.

## 2. Materials and Methods

### 2.1. Data Source

The data for this study come from the “Spanish Pharmacovigilance Adverse Reactions Data” (FEDRA) of the “Spanish Pharmacovigilance System for Medicinal Products for Human Use” (SEFV-H), managed by the “Spanish Agency for Medicines and Health Products” (AEMPS). SEFV-H gathers data from the 17 autonomous pharmacovigilance centers plus the AEMPS. Each Autonomous Community is responsible for evaluating and registering the adverse effects suspected from the use of a specific drug in a FEDRA database [24,25]. 

The FEDRA is a passive reporting system that relies on individuals sending reports about their experiences to SEFV-H. It is designed to allow the detection of unusual or unexpected patterns of adverse event reporting, not for determining if a vaccine or drug causes a health problem.

The information collected in the FEDRA database comes from a wide array of sources, including patients and parents, state health agencies, pharmacies, health care providers, and the makers of the drugs including vaccines [25], who complete each AEFI report. 

In the Valencian Community since 2000, all immunizations events have been recorded in the Vaccination Information System (SIV), including AEFI reports [26], and since 2005 that system has been a population-based individual register linked to the FEDRA, and only health care workers have access to this platform. All AEFI reports are evaluated and uploaded to the FEDRA by technicians from the Valencian Community Pharmacovigilance Center.

### 2.2. Data Extraction

The FEDRA and SIV reporting forms include information about the event, collecting data such as time of onset following immunization, outcome, hospitalization dates, patient identifiers (age, sex), and the vaccine characteristics (type, name, and administration route), vaccine antigens, vaccination date, and dose number. Typically, each AEFI report lists several symptoms, signs, and diagnoses that had been recoded from the reporter’s description into standardized terms using the Medical Dictionary for Regulatory Activities (MedDRA® ) [27].

We received SEFV-H (AEMPS) authorization to analyze the FEDRA database regarding the HPV vaccine in the Valencian Community from 1 January 2008, through 31 December 2018. The results, discussion, and conclusions of this paper show the authors’ point of view, and they do not represent in any way the position of the SEFV-H or the AEMPS regarding this issue.

### 2.3. Descriptive Analysis

Rates of AEFI reports per 100,000 doses of HPV vaccine administered among the population of interest over the period between 1 January 2008 and 31 December 2018 were calculated. The denominator data utilized to estimate AEFI reporting rates were obtained from the national HPV vaccine coverage survey for ages 11–49 years old. The rates of AEFI reports were also analyzed by age group at the time of vaccination: 11 to 13 years old, 14 to 15 years old, 11 to 15 years old, and <15 years old.

The confidence intervals for the AEFI reporting rates were calculated by assuming a Poisson distribution and by applying the modified Wald method [28] if the rate numerator was zero.

To analyze the additional diagnoses or symptoms listed in more than one percent of AEFI records (e.g., headache, dizziness), additional terms were created. Reaction terms listed in less than one percent of records were grouped into broader categories based on the organ system where the reaction was manifested (e.g., gastrointestinal, neurological).

### 2.4. Trend Analysis

A joinpoint regression analysis was used to determine time segments and time points in AEFI trends for each age group. Each joinpoint (if any) showed a significant change in trend and an annual percentage of change (APC) of the rates, and the corresponding confidence interval at 95% (CI 95%) was computed from each trend segment. A negative APC indicated a decreasing trend. The year 2008 was excluded from these analyses, since the detection of a point of change in the trend was substantially affected by this year’s data. To build the model, the National Cancer Institute’s freeware Joinpoint Regression Program V 4.7.0.0 was used [29], under the usual assumption of heteroscedastic variances for the rates. The weighted least square method was used to estimate the model. The Monte Carlo permutation test [30] was used to prove the existence of statistically significant joinpoints (*p* < 0.05). This test is usually used in studies of rate trends.

### 2.5. Ethical Statement

The data collected in the FEDRA are monitored by the SEFV-H and managed by AEMPS. SEFV-H consolidates the health administration activities, which are executed permanently to collect, elaborate, and process the information about suspicions of adverse reactions to identify unknown risks or changes in known risks. Further, the SEFV-H manage studies as is deemed necessary to confirm or quantify said risks [24]. Finally, AEFI reporting is a routine surveillance program which does not require informed consent and uses depersonalized, de-identified data.

## 3. Results

Between 1 January 2008 and 31 December 2018, 664,722 bHPV, qHPV, and nHPV vaccinations were administered in the Valencian Community according to the Vaccination Information System (SIV) [20].

A total of 812 adverse events were recorded in the SEFV-H database (FEDRA), corresponding to 317 individual AEFI reports, with a mean of 73.81 (SD 84.31) per year; the minimum was 10 in 2017 and the maximum 296 in 2009. There were 2.57 events per AEFI report. The overall AEFIs reporting rate was 47.54 notifications per 100,000 vaccine doses distributed (Table 1). 

The AEFI reports concerned 65.51% qHPV, 34.18% bHPV, 0.32% nHPV; girls aged between 12 to 17 years old reported 94.45% of AEFIs. The primary reporters were submitted by health professionals (98.48%), and only 1.5% were submitted by patients or their lawyers.

### 3.1. Trends over Time by Setting

**Figure 1:**
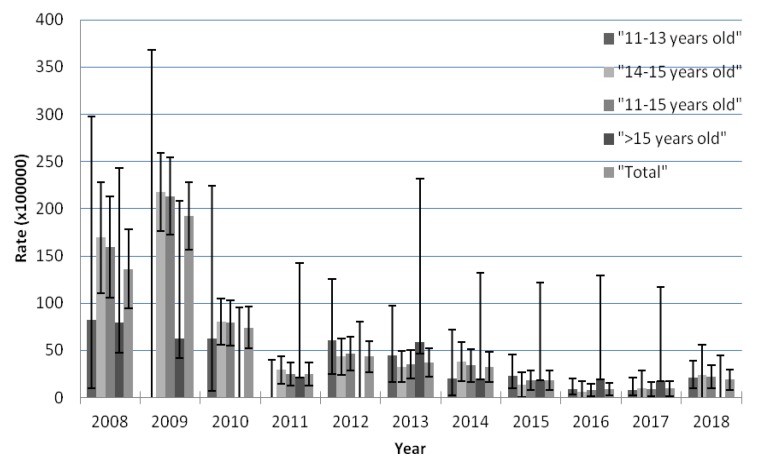
AEFI reporting rates per 100,000 by age group in the Valencian Community, Spain (2008–2018).

Throughout the study period (2008–2018), we observed that forty-seven percent (47.78%, *n* = 151/317) of the total AEFI-reports was received mainly in 2008 and 2009, the first years of the HPV vaccination program in the Valencian Community. The AEFI reporting rate in 2009 was the highest among girls aged 14 to 15 years old (217.75 per 100,000 doses [CI 95% (176.1–259.4)]; this group also received the greatest number of vaccinations. Regarding the overall population-based reporting rate per 100,000 doses, we found a decreasing trend from 2009 (192.2 [156.28–228.11]) to 2017 (9.46 [1.89–17.04]). Figure 1 shows the reporting rates of AEFI reports following immunization per 100,000 by age group.

### 3.2. Trend in AEFI Notifications by Age According to the Joinpoints Identified by the Analysis

We observed a decline in the AEFI reporting rates for HPV vaccines; there was one significant joinpoint in 2011 (*p* = 0.022), and two periods were identified. The first period, between 2009 and 2011, has a decreasing trend (−54.9% annual change, 95%CI [−75.2; −17.7]), and it was followed by a flat, plain trend between 2011 and 2018 (−13% APC, 95%CI [−26.1; 2.4]), with a nonsignificant decrease. 

**Figure 2:**
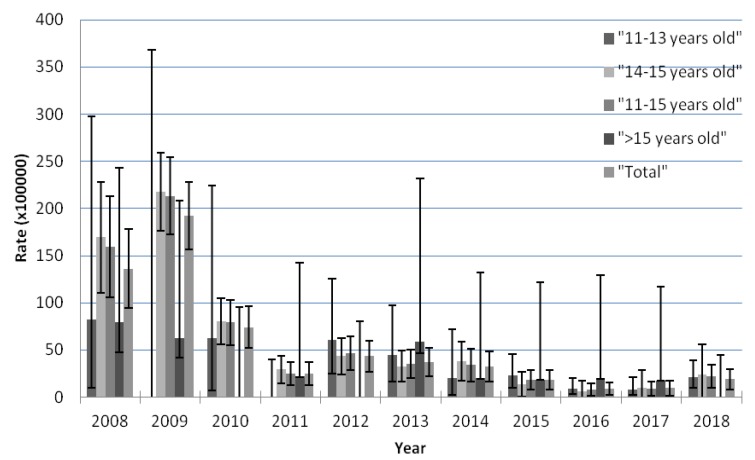
Trends in AEFI reporting rates following HPV vaccines in the Valencian Community, Spain, according to the joinpoints identified by the analysis by different age groups. (**a**) 11–13 years old group, no joinpoint was detected; (**b**) 14–15 years old group, a joinpoint was detected in 2011; (**c**) > 15 years old group, no joinpoint was detected; (**d**) for all the groups, a joinpoint was detected in 2011.

We analyzed the APC and joinpoints separately according to different age groups but found no joinpoints in either the 11–13 years old and older than 15 years old groups. However, one significant joinpoint was identified in the 14–15 years old group in 2011 (*p* = 0.006), leading to two periods with different trends; the fast descending trend in the AEFI reporting rates was in the 2009–2011 period, with −58.4% (95%CI [−73.9; −33.8]) APC, while the trend in the second period, 2011–2018, was −8.8% (95%CI [−27.7; 15]) APC. The fast descending trend in the rates observed in the group as a whole could be explained by the AEFI reporting rate trend of girls aged 14–15 years old. Results from selected age groups are displayed in Figure 2. (See Table A1).

### 3.3. The Trend of AEFIs Reported as ‘Serious’

The rate of serious events was calculated using the total number of AEFI reports with some adverse events classified as serious, and the number of doses as the denominator. A total of 83 AEFI reports (26.18%) from 317 were recorded as serious events, and more than 20% (65/317) were generated in the first three years of the HPV vaccination program in the Valencian Community. The rate of serious AEFI reports for HPV vaccines was 12.49 per 100,000 doses (Table 1).

In the period of the study, there were 1.14 serious events per AEFI report, 44.58% (362/812) were reported as a serious adverse event, and about thirty percent (29.8% (242/812)) were recorded as recovered or recovering at the moment of reporting. Not a single death was recorded in eleven years of surveillance. 

**Figure 3:**
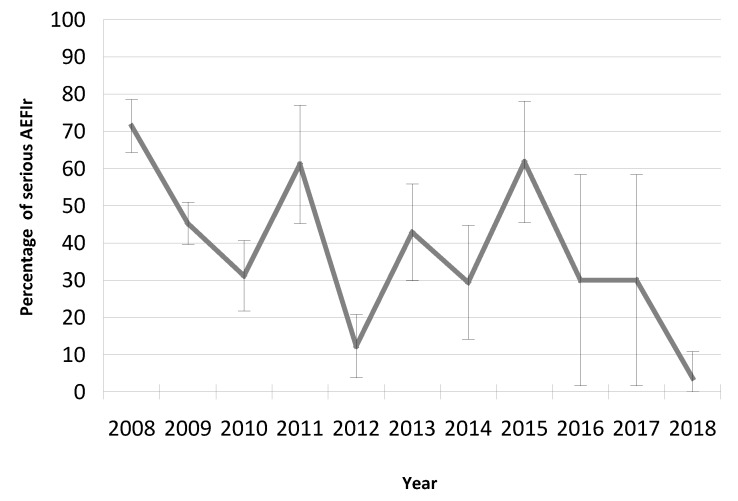
Trend of the percentage of serious AEFI reports in relation with the total AEFI reports in the Valencian Community, Spain (2008–2018).

Figure 3 shows the trend of the percentage AEFIs reported as serious overall AEFI reports. From 2008 to 2018 in the Valencian Community, the percentage of serious events for the vaccines administered declined in 2018 as compared with the earlier years, following reductions in the number of AEFIs notified. Although the global trend of AEFIs reports fell, the trend of the percentage of AEFIs reported as a serious event seems to remain nearly constant.

### 3.4. Events Following Immunization Reactions

Table 2 shows the top 11 AEFIs for HPV vaccines. The most frequently reported adverse events were injection site reaction (10.1% of 812 AEFI records) followed by headache (9.7%), dizziness (8.8%), and fever (7.6%). One case was reported as disability with deafness without information related to recovery, there was a case of bronchospasm, another with optic neuropathy, and one case of hepatitis, none with information of recovery or surveillance.

## 4. Discussion

Pharmacovigilance is a critical component of healthcare for determining the benefit to risk ratio of treatment and the monitoring of vaccine safety in order to ensure patient and parent trust. As expected when introducing a new vaccine, the AEFI reporting rate for HPV vaccine was very high in 2009 (192.2 per 100,000 doses), and the highest annual number of cases (*n* = 110; 34.7%) was found in the Valencian Community during the first 10 years of this vaccine’s administration. Historical data in some countries [31,32,33] show that initial high levels of AEFI reporting occur each time a new vaccine is introduced, as immunization providers are more likely to report milder, less serious AEFIs for vaccines they are not familiar with, followed by a reduction and stabilization of reporting over time (Weber effect) [34]. This enhanced propensity to report events following newer vaccines increases the sensitivity of the system for detecting signals of serious, rare or previously unknown events.

Current data held by the FEDRA in the Valencian Community indicate that the annual number of reports decreased substantially in the following 10 years (from 110 AEFIs reports in 2009 to 12 in 2018).

There are some reasons that can explain this reduction and stabilization trend of AEFI reporting. First, the Weber effect could be potentiated by the two cases reported in February 2009 (early years of the new HPV vaccine). Those cases came to the attention of the media and resulted in considerable media interest both locally and internationally [21,22], which could have affected the following years’ AEFIs report ratio until 2011, when a change in trend occurred, as it is in this year that the health authorities changed the qHPV vaccine for the bHPV vaccine [14,17]. 

The change in the cohort age from 14-year-olds to 12-year-olds of the Valencian Community vaccination program could have decreased the AEFI reports that were mainly related to anxiety, as shown in a systematic review by Loharikar et al. [35], which described a high incidence of anxiety-related AEFI clusters in adolescents. 

Currently, the acceptance for the HPV vaccine is high [36], and the reports related to the controversy in which it was involved during the first years of the vaccination program have decreased. This more lenient social environment could have an influence on the risk perception of the potential informers when faced with an AEFI.

The trend analysis of the AEFI reporting rate from 2008 to 2018 showed a drop with a joinpoint in 2011, and during the first three years (2008–2010), the rate was around 100 AEFIr per 100,000 doses, as described in Slovenia [37] and the UK [38],but higher than in other countries in the early years of the vaccination program [33,37,39,40,41,42,43,44]. However, since 2011, the trend has seen a stabilized period with little APC, and currently, in the Valencian Community, the AEFI reporting rate after HPV immunization is low and consistent with rates of events seen elsewhere and in different regions [43,45,46,47].

The rate of reports submitted declined in 2016 and 2017 but slightly increased in 2018, although more years of monitoring are needed in order to evaluate if this could represent a new joinpoint.

In our study, the rate for serious AEFIs was also higher in the first three years in the Valencian Community (more than 20 AEFIr per 100,000 doses), although this rate was slightly higher than in other countries [37,47]. However, the exposure by the press could have contributed to this phenomenon [48,49]. The last few years seemed to have had a more stable situation, with a reporting rate similar to those of other countries [41,46,50,51]. 

The AEFIs most commonly reported, which were milder in nature (such as headache, dizziness, myalgia, and malaise), are consistent with those of other studies [33,37,41,44,46,47,52,53,54] and usually related or accompanied by anxiety symptoms [37,46,55]. On the other hand, the emotional component of the adolescent population (fear, anxiety) when facing vaccination is known [35,56,57], which could have had a negative impact on the vaccination process. 

In the analysis according to age groups, we found that the AEFI reports rate was influenced by the 14-15-year-old adolescent girls, with this group being the largest recipient of HPV vaccines, which determined the trend change in 2011. Additionally, until that year, the vaccination was carried out mainly in schools, and the media effect of the cases in 2009 had remained in the collective memory. Further, in 2011, the change in the type of vaccine took place. In our study, we could not verify if the vaccination center (school or health center) influenced the AEFI reporting rate. We believe that the fear of HPV vaccination, a common experience among adolescents [56,58], could have increased the AEFI reporting rate. 

This study does not exclude the presence of rare AEFI following HPV vaccination, such as complex regional pain syndrome (CRPS), postural orthostatic tachycardia syndrome (POTS), and chronic fatigue syndrome (CFS), because our data come from a passive reporting system and the information in general are disaggregates, with these diagnoses being generally poorly understood and under-recognized. Further, a larger sample is needed for performing a cluster analysis [53,54].

The major limitation of a passive surveillance system such as the one available in the Valencian Community is that it can only identify early warning signals and neither can it estimate the risk related to an unexposed population nor does it exclude risks with a good degree of certainty [41,59]. Moreover, the diagnoses in our study were not validated by a review of the patient records because this information was not made available for review; therefore, we were unable to validate AE reports independently. The FEDRA accepts all reports without judging whether the event was caused by the vaccine [25]. 

The AEFI reporting ratio is useful for benchmarking and following trends over time; however, this ratio does not provide information on the quality of the reporting system and does not guarantee the capacity to detect and manage a vaccine safety problem at a national level [60]. However, this analysis allows us to recognize the possible impact of changes in the strategies of a national/regional vaccination program. Additional efforts are required to ensure and improve data quality, AEFI reporting, and surveillance of immunization safety in our community.

## 5. Conclusions 

The trend on AEFI reporting rate after HPV immunization in the Valencian Community comprised two periods, an earlier one with a faster decrease followed by a stable period, explained only by the AEFI reporting rate in the group of girls aged 14–15 years old. Currently, the AEFI reporting rate shows a decreasing trend following a possible Weber effect and could be affected by media attention and coverage. The health authorities should improve the training of the potential users of the system of declaration of adverse effects in order to avoid ambiguous terms and to increase the quality of the reports.

## Figures and Tables

**Table 1:** Adverse events following immunization (AEFI) reporting rate following human papillomavirus (HPV) immunizations in the Valencian Community, Spain (2008–2018).

Years	AEFIr	Doses	AEFIr Rate ^1^	95%CI	Total AE	AEFIr with Serious AE	Total Serious AE	Total AE/AEFIr	Total AE Serious/AEFIr	AEFIr Serious Rate ^1^
2008	41	30,112	136.16	94.48–177.84	154	18	110	3.76	2.68	59.78
2009	110	57,233	192.2	156.28–228.11	296	24	134	2.69	1.22	41.93
2010	43	58,201	73.88	51.80–95.96	93	13	29	2.16	0.67	22.34
2011	17	68,089	24.97	13.10–36.84	36	6	22	2.12	1.29	8.81
2012	27	62,257	43.37	27.01–59.73	57	3	7	2.11	0.26	4.82
2013	24	64,126	37.43	22.45–52.40	56	8	24	2.33	1.00	12.48
2014	16	49,019	32.64	16.65–48.63	34	2	10	2.13	0.63	4.08
2015	13	70,180	18.52	8.45–28.59	34	6	21	2.62	1.62	8.55
2016	7	78,761	11.04	2.30–15.47	15	1	1	1.43	0.43	1.27
2017	7	63,402	9.46	1.89–17.04	10	1	3	1.67	0.50	1.58
2018	12	63,342	18.94	8.23–29.66	27	1	1	2.25	0.08	1.58
Total	317	66,4722	47.54	42.30–52.78	812	83	362	2.57	1.14	12.49

^1^ Reports per 100 000 doses distributed; AEFIr: Adverse events following immunization report; AE: Adverse event.

**Table 2:** Distribution and frequency of reactions listed in AEFI records.

	2008	2009	2010	2011	2012	2013	2014	2015	2016	2017	2018
AEFI ^a^	(*n*)	(*n*)	(*n*)	(*n*)	(*n*)	(*n*)	(*n*)	(*n*)	(*n*)	(*n*)	(*n*)
Injection site reaction ^b^	8	24	11	6	9	4	2	1	6	3	8
Headache	7	33	6	4	7	11	3	5	2	0	1
Dizziness	12	32	7	2	0	3	6	4	1	1	4
Fever	2	26	12	6	7	9	2	3	1	2	0
Syncope	11	21	12	3	5	2	3	3	1	0	1
Myalgia and malaise	1	16	6	2	4	3	1	6	1	2	1
Nausea/ vomiting	4	20	4	1	3	4	3	1	0	0	1
Presyncope/Pallor	6	11	2	1	2	0	3	1	1	1	5
Hypersensitivity reaction ^c^	3	11	5	0	6	1	2	1	0	1	1
Seizure	0	9	3	0	1	2	0	0	0	0	0
Somnolence	3	4	2	1	1	0	0	1	0	0	1
Others	97	89	23	10	12	17	9	8	2	0	4
Total	154	296	93	36	57	56	34	34	15	10	27

Abbreviations: AEFI, adverse event following immunization; ^a^ using MedDRA terms. More than 1 code may be assigned to a single report; ^b^ local injection site reaction MedDRA codes include injection site abscess, injection site abscess sterile, injection site atrophy, injection site cyst, injection site desquamation, injection site hemorrhage, injection site hypersensitivity, injection site inflammation, injection site mass, injection site necrosis, injection site nodule, injection site edema, and injection site pain; ^c^ hypersensitivity reaction MedDRA codes include anaphylactic reaction, anaphylactic shock, anaphylactoid reaction, cross-sensitivity reaction, dermographism, hypersensitivity, urticaria, urticaria thermal, and urticaria vesicular.

## Appendix A

**Table A1:** Trends in AEFI reporting rate following HPV immunizations in the Valencian Community, Spain according to age group.

Years	11 to 13 Years Old				14 to 15 Years Old				>15 Years Old *			
	ICSR	Doses	AEFI Rate	95%CI	ICSR	Doses	AEFI Rate	95%CI	ICSR	Doses	AEFI Rate	95%CI
2008	2	2424	82.51	9.99–298.05	32	18,875	169.54	110.8–228.28	7	8813	79.43	31.93–163.65
2009	0	1003	0	0.00–367.78	105	48,220	217.75	176.10–259.4	5	8010	62.42	20.27–145.67
2010	2	3217	62.17	7.53–224.58	41	51,121	80.2	55.65–104.75	0	3863	0	0.00–95.49
2011	0	9130	0	0.00–40.40	16	54,350	29.44	15.01–43.86	1	4609	21.7	0.55–120.89
2012	7	11,494	60.9	24.49–125.48	20	46,171	43.32	24.33–62.30	0	4592	0	0.00–80.33
2013	6	13,418	44.72	16.41–97.33	15	45,625	32.88	16.24–49.51	3	5083	59.02	12.17–172.48
2014	2	10,083	19.84	2.40–71.65	13	33,958	38.28	17.47–59.09	1	4978	20.09	0.51–111.93
2015	8	34,885	22.93	9.90–45.19	4	29,914	13.37	0.27–26.48	1	5381	18.58	0.47–103.54
2016	5	56,405	8.86	2.88–20.69	1	17,283	5.79	0.00–17.13	1	5073	19.71	0.50–109.83
2017	4	47,565	8.41	2.29–21.53	1	10,241	9.76	0.00–28.90	1	5596	17.87	0.45–99.56
2018	10	46,685	21.42	10.27–39.39	2	8475	23.6	0.00–56.31	0	8182	0	0.00–45.09
Total	46	236,309	19.47	13.84–25.09	250	364,233	68.64	60.13–77.15	20	64,180	31.16	17.50–44.82

* 95% confidence interval. In women aged over 15 years old, the limits have been calculated exactly (except for the period as a whole).
